# Current Clinical Practice for the Use of Hypnotics to Manage Primary Insomnia in Adults in a Tertiary Hospital in Saudi Arabia: An Audit Study

**DOI:** 10.3390/pharmacy7010015

**Published:** 2019-01-26

**Authors:** Ali Dobia, Kath Ryan, Daniel Grant, Ahmed S. BaHammam

**Affiliations:** 1 School of Pharmacy, University of Reading, Berkshire RG6 6AP, UK; k.m.ryan@reading.ac.uk (K.R.); d.t.grant@reading.ac.uk (D.G.); 2 University Sleep Disorders Center, College of Medicine, King Saud University, P.O. Box 225503, Riyadh 11324, Saudi Arabia; ashammam2@gmail.com; 3 The Strategic Technologies Program of the National Plan for Sciences and Technology and Innovation, Riyadh 11633, Saudi Arabia

**Keywords:** clinical practice, hypnotics, benzodiazepines, z-drugs, audit, primary insomnia, tertiary hospital, Saudi Arabia

## Abstract

Despite the risks associated with hypnotics and their recent increased use in Saudi Arabia, there are no specific national guidelines for using these medicines to treat insomnia nor are there any data on how these medicines are currently prescribed. There is the potential, however, that some physicians might be adhering to the United States guidelines. The current audit study was aimed to assess the current practice in treating insomnia with hypnotics in Saudi Arabia, and to evaluate its agreement with the US guidelines. The audit was conducted using data collected between April 2012 and March 2017 at King Fahad Central Hospital (KFCH; Jazan), of patients who were either prescribed benzodiazepines (BZDs) or Z-drugs or diagnosed with insomnia. The audit criteria followed two US guidelines for the management of insomnia in adults. Data included documented diagnosis, use of CBT-I (Cognitive Behavioral Therapy for Insomnia), use of BZDs and Z-drugs including treatment regimen, and whether physicians prescribed anti-histamines for insomnia. The data were analyzed using STATA 14 after transcription to a MS XL file. Of the 504 records reviewed, 379 patients (75%) were prescribed BZDs or Z-drugs; only 182 (48%) of them had clearly documented indications for their use. Three hundred and seven patients (60%) were diagnosed with insomnia; none of them received CBT-I as initial treatment. No patients on long-term use of hypnotics were reviewed by their physicians after they began using the medication. More than 43% of patients were prescribed anti-histamines for insomnia. No records met all (or even six) of the seven criteria. KFCH physicians do not follow US guidelines. Therefore, the Ministry of Health (MOH) should improve its administrative systems including documentation, and instead of using international guidelines that are seldom followed, physicians should be trained in prescribing hypnotics and national guidelines need to be developed.

## 1. Introduction

Studies show that while benzodiazepines (BZDs) and Z-drugs (eszopiclone, zolpidem and zaleplon) are associated with a high risk of patient misuse, and cause addiction, dependence, falls, accidents, cognitive impairment and withdrawal symptoms, the therapeutic benefits outweigh the disadvantages if the drugs are prescribed appropriately [1,2]. In the United States, the United Kingdom, and other countries, the known risks have led to clinical guidelines to help physicians prescribe the medicines safely, and to minimize the risk of addiction [3,4,5,6,7].

While these guidelines contain attributes specific to the countries where they are applied, there are recommendations common to all, among which is the importance of using the smallest dosage possible to induce a therapeutic effect. Other shared recommendations include the preferred length of the prescription (2–4 weeks), the consideration of non-pharmacological treatments prior to hypnotic prescription, using drugs with the lowest cost, the recommendation that new medicines be withheld if the prescribed hypnotics are unsuccessful (except when the patient experiences adverse effects), and that prescriptions must include a withdrawal plan [3,6,7,8].

BZDs and Z-drugs have severe adverse effects if taken for longer than the recommended treatment period (more than 4 weeks) [3]. Despite this, the effectiveness of these medicines to treat insomnia justifies their administration if they are used appropriately and for short durations. The national guidelines for insomnia management in the US and UK begin with sleep hygiene, non-pharmacological remedies such as cognitive behavioural therapy for insomnia (CBT-I), and only after these treatments have been attempted, is the short-term use of hypnotic medicines considered for patients with severe insomnia [3,6,7]. Even when the guidelines are followed, the literature shows that patient misuse of BZDs and Z-drugs still occurs [9,10,11,12]. In addition, prescribing hypnotics for long-term use might indicate that physicians do not adhere to the guidelines.

Some audit studies have been conducted on the use of hypnotics in different regions across the world. One such study used 308 medical records to assess the use of hypnotics to manage insomnia, and compared the results to the National Institute of Clinical Excellence (NICE) guidelines. The study found that a non-pharmacological treatment was provided to only 25% of patients, whereas 33% were prescribed hypnotics for less than one year and 10% were prescribed these medicines for more than 10 years [11]. Another prospective audit study was conducted in Ireland to assess the level of prescription compliance using the main components for Z-drugs and BZD prescribing guidelines. The study over four weeks involved 81 community pharmacies and reported that less than one-fifth of the prescriptions were fully compliant with the assessment criteria; many of the prescriptions also had discrepancies [12]. 

Although pulmonologists in Saudi Arabia began sleep studies in the early 1990s [13], the Saudi Commission for Health Specialties (SCHS), which authorizes all health specialty professionals in Saudi Arabia, did not recognize sleep medicine as an independent specialty until 2012 [14]. For this reason, there is insufficient data concerning insomnia treatments in the Kingdom of Saudi Arabia (KSA), including how BZDs and Z-drugs are used in the Kingdom. Even though these types of medicines are restricted in Saudi Arabia and only specialists and consultants are authorized to prescribe them, most of the literature considers BZD usage for psychiatric disorders only [15,16,17]. Thus, further studies are necessary to investigate the use of these medicines for insomnia in the KSA. The motivation to conduct this audit study, in addition to the aforementioned problems, included the absence of well-developed clinical guidelines for the use of BZDs and Z-drugs to manage primary insomnia in the KSA. Thus, this audit study reports data gathered on the current clinical practice in a major regional Ministry of Health (MOH) referral hospital (King Fahad Central Hospital (KFCH) in Jazan) where hypnotics are used to treat insomnia. 

This audit aims were to assess the current practice to treat insomnia with hypnotics in Saudi Arabia, and to evaluate its agreement with the US guidelines. There are two such guidelines, one from the American Academy of Sleep Medicine (AASM) and the other from the American College of Physicians (ACP) [3,7]. In this audit study, we attempt to answer the following questions in the absence of national clinical guidelines: what is the current clinical practice when prescribing hypnotics for patients with insomnia in Saudi Arabia, and how different is the current practice from the American guidelines? The US guidelines were used because there are no country-specific guidelines for the treatment of insomnia in Saudi Arabia, and most Saudi physicians were taught using the American system at medical schools. In addition, the Saudi MOH and other departments that provide most medical services in Saudi Arabia often adopt US guidelines [18] because they are believed to be the best medical practice [19]. 

After the research criteria were developed, a systematic review of randomized controlled trials (RCT) to elucidate the usefulness of pharmacological agents to treat chronic sleep disorders from the AASM was published [4]. The review focused only on treatment rather than other management criteria, and it was recommended that treatments should follow the 2008 guidelines of the AASM [4]. This did not affect the previously developed criteria. The present paper reports on the first four steps of the traditional audit: it identifies the problem, develops the tool to measure the outcome, collects data and compares the findings with established standards, and suggests changes [20]. 

## 2. Materials and Methods 

The study was conducted at KFCH, a 500-bed tertiary hospital in Jazan that serves 20 other hospitals in the region with approximately 1.5 million inhabitants. A tertiary hospital is defined as a facility that provides specialized care using highly advanced and complex procedures performed by medical specialists [21]. These facilities usually deal with referrals from primary and secondary care facilities. Thus, the audit was conducted in this facility because in Saudi Arabia these types of medication cannot be prescribed in primary care since prescription of controlled drugs is limited to secondary and tertiary care centers.

Since 2009, KFCH has used MedicaPlus, an electronic information system which also permits electronic prescriptions. MedicaPlus connects the hospital’s various departments. Information can be retrieved using patient name, ID numbers, diagnosis, and prescribed medicines. Audit criteria were developed from the AASM and ACP clinical guidelines [3,7] as they are the two published guidelines most often followed; this means the criteria have already been validated. The data collection tool was also verified by an expert in clinical audits (D.G.), as well as the researcher’s supervisor, prior to the performance of the study. All standards and consensus recommendations related to the use of BZDs or Z-drugs and/or the management of primary insomnia from the guidelines were incorporated into the criteria to develop a data collection tool (Table 1) to be applied in the KSA. The criteria were devised by the authors to indicate the best practice for insomnia treatment. Standards and consensus recommendations that were not related to the management of primary insomnia, for example diagnosing insomnia, were excluded. Thus, the data collection audit tool was designed to retrieve patients who had been:

- not only prescribed BZDs or Z-drugs but also had clear documentation of their use;
- given CBT-I as the initial treatment (documented);
- prescribed these drugs when physicians decided to use pharmacological treatment;
- initially prescribed the lowest licensed dosage of BZDs or Z-drugs;
- prescribed the drugs specifically for the maximum recommended duration (4–5 weeks);
- on long-term use of the medicines and were reviewed by their doctors every few weeks or on a monthly basis, and then every 6 months (documented).

During the study period of March–May 2017 at KFCH, the first author (A.D.) performed an electronic search of the medical records using the terms insomnia and/or the generic and trade names of BZDs and Z-drugs, with the advice of the hospital pharmacy and quality management departments, to identify patients. After eliminating duplicates, 771 records met the inclusion criteria of adult patients (aged 18 years and over) with insomnia who were prescribed drugs as a treatment, or their diagnosis was documented, during the period from April 2012 to March 2017. Data were collected from medical records of patients admitted to different wards, as well as for outpatients. Patients were excluded if they were prescribed any of these medicines for other indications such as an add-on therapy for epilepsy, dementia, psychosis or other psychiatric disorders; as part of substance withdrawal treatment (e.g., methadone or alcohol); for terminal illness; or if patients were admitted to the intensive care unit (ICU). After applying the exclusion criteria, 504 patients were deemed appropriate for the data collection process.

As this study is one of three studies of a PhD research project at the University of Reading in the UK, the University of Reading Ethics Committee (UREC) provided guidance and approved the study number (17/15), on 8 March 2017. On 19 March 2017, similar guidance and approval were given by the Research Ethics Committee of the General Directorate of Health Affairs (Jazan), Ministry of Health, Saudi Arabia. Permission to access the data was obtained from the Hospital Director and coordinated with the medical records department.

Before data collection began, the researcher received suitable training from the medical records technicians on how to use medical records and collect the required data at the hospital. The data collection tool was piloted using 10 medical records to ensure that all relevant information could be gathered. Obtained data were anonymized, and the last four digits of each patient’s hospital ID substituted with identification letters. Names, addresses or other personal information were not collected. In addition, the prescribers’ identities were anonymized using a unique identifier number. Information on KFCH physicians’ current practices was gathered from the medical records and entered into the data collection tool for comparison with the audit criteria. No names, addresses and other personal information were collected.

**Figure 1:**
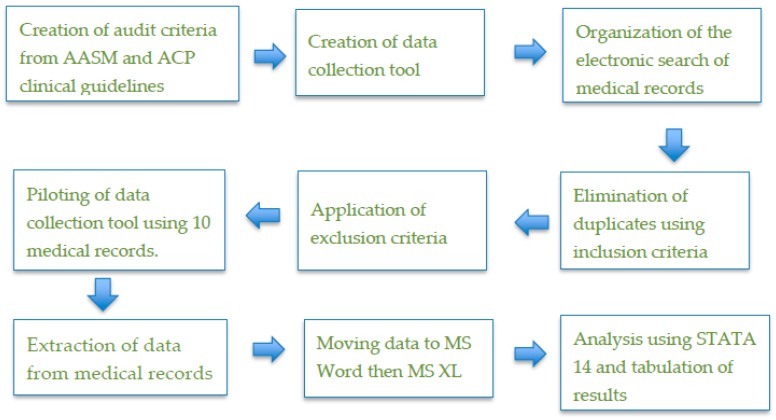
Summary of the study process.

Data extracted from the medical records were coded by one of the authors (A.D.) as “YES or NO” options to indicate whether or not the criterion had been met. Data were transferred from the data collection sheet (MS Word) into an MS XL file and then entered into STATA 14 for analysis [22]. All variables (i.e., criteria) had a “YES/NO” response. These responses were recoded for convenience as follows: all “YES” responses were recoded as 1, and all “NO” responses were recoded as null. Tabulate (tab) and summarize (sum) commands provided the descriptive statistics in STATA and showed the number (and percentage) of records that met each criterion. The reliability of the findings was addressed by using a structured and documented process for data collection and analysis. The validity was addressed by the process of inspecting the data collected at the different stages. Figure 1 presents a summary of the study process.

## 3. Results

The results were computed based on 504 medical records of patients treated for insomnia using BZDs or Z-drugs or any other medication. Of the 504 records which met the inclusion criteria:

- 75% (379) of patients were prescribed BZDs or Z-drugs, and of these, 48% (182) had clearly documented indications for their use.
- 61% (307) of patients received some form of medication for insomnia; none were offered or received CBT-I as a first-line treatment; 59% (182) used BZDs or Z-drugs as first treatment, of which 65% (118) began with the lowest recommended dose; 51% (93) of all prescriptions complied with the recommended duration.
- After being prescribed BZDs or Z-drugs and beginning medication use, no patient was reviewed by the physician for the effects of long-term use.
- 44 % (134) of patients were given anti-histamines or anti-histamine analgesics (combination of paracetamol and diphenhydramine) for insomnia.

In addition, antipsychotic medicines such as quetiapine and olanzapine or antidepressants such as mirtazapine and doxepin were prescribed for some patients as first line pharmacological treatment. Table 2 shows that physicians in KFCH do not follow the current US guidelines for insomnia treatment.

The most important points are that CBT-I was not used as an initial treatment, BZDs and Z-drugs were prescribed without documented indications for use, patients were not reviewed by their doctors once they started using the medicines, and physicians used non-recommended medicines such as anti-histamines to treat insomnia. Table 3 shows how many criteria were followed based on each individual record.

It should be noted that none of the records met all the criteria or even six of them, and only 11 (3.6%) met five criteria. Approximately one-third of the records of patients diagnosed with insomnia met only one criterion. This means that physicians at KFCH are either unaware of current guidelines or are unwilling to comply with them when treating patients with insomnia.

## 4. Discussion

To the best of our knowledge, this audit study is the first to assess the practice of prescribing BZD and Z-drugs in the KSA. The current results indicate that physicians at KFCH do not follow the US guidelines for prescribing BZD or Z-drugs for insomnia, nor do they comply with all audit criteria developed from these guidelines. After excluding the first and sixth criteria (using CBT-I as initial therapy and reviewing patients taking the drugs for long periods), only 60 medical records met the remaining five audit criteria (less than one-fifth of cases). Additionally, 109 medical records of the 307 patients who were diagnosed with insomnia failed to meet even one of the six criteria. Therefore, physicians treated one-third of patients with primary insomnia without reference to the guidelines. This suggests that many KFCH physicians are either unaware, unwilling or unable to comply with international guidelines. 

Furthermore, documented indications of use were found in less than half of patients prescribed the drugs under investigation. This might be due to an inadequate prescribing system, physicians’ busy schedules, or because they are not aware of the guidelines or the value of recording indications for drug use. Alternatively, physicians might not believe that the symptoms/indications warrant documentation. However, it is important for other physicians to have a complete patient history of drug-use to ensure that they can undertake a thorough diagnosis of the patient before initiating or continuing treatment [23].

The Clinical Guidelines for the Evaluation and Management of Chronic Insomnia in Adults [3] recommend that BZDs and Z-drugs should be prescribed only after accurate diagnosis and when symptoms are associated with daily dysfunction [3]. Telephone interviews in the UK, Germany, Italy, and Portugal have found that chronic insomnia is related to anxiety and depression [24]. For this reason, a clear indication should be documented to improve physician-to-physician communication and help physicians to consider the underlying issues associated with chronic insomnia. To avoid prescription duplication, physicians need to document indications for the use of BZDs and Z-drugs, which would assist other professionals with patient treatments. Furthermore, poor documentation can lead to severe consequences such as misdiagnosis, lack of communication between physicians, and/or inappropriate prescribing of medications [25]. 

Our study findings show that compared to US guidelines, BZDs and Z-drugs were prescribed inappropriately for the majority of patients at KFCH. The audit also revealed that no patient received CBT-I as a preliminary treatment step, as recommended by the US guidelines [3,7]. Although the evidence shows that CBT-I has the same effect in the management of insomnia as pharmacological treatments for short periods, is superior for long periods, and is preferred by patients [26], this was clearly not the case at KFCH where patients did not receive CBT-I. 

The guidelines from the AASM state that combined treatments (CBT-I and pharmacological) are required and even preferred when the availability of other treatments, the occurrence of side effects, dependence on past treatment, and patient preference are considered [3]. This audit found that pharmacological treatment was the only documented method of treatment administered to patients with insomnia at KFCH. This discrepancy might be due to a belief that CBT-I is not effective or worth documenting, or due to the physicians’ busy schedule and the short consultation time. The lack of a referral process or shortage of psychologists with expertise in CBT-I might also explain its non-use. According to Saudi Arabia–World Health Organization (2014), in the Kingdom (population: 32 million) only 1.38 psychologists exists per 100,000 of the population [27].

The results also indicated that physicians at KFCH did not prescribe BZDs or Z-drugs for two-fifths of patients diagnosed with insomnia. The audit showed that physicians prescribed antipsychotic medicines such as quetiapine and olanzapine or antidepressants such as mirtazapine and doxepin. In addition, many prescribed anti-histamines or anti-histamine analgesics, either alone or with other medicines, were given to more than 43% of patients, even though the AASM recommends neither for chronic insomnia [3]. Although the KSA’s Ministry of Health Drug List recommends the use of temazepam and nitrazepam [28], physicians at KFCH do not prescribe these medications. These findings confirm that many physicians at KFCH do not comply with the US guidelines concerning the use of BZDs or Z-drugs as an initial treatment for insomnia. This might be due to unawareness or a lack of concern regarding side effects. 

More worryingly, over one-third of patients were not prescribed the lowest effective dose of BZDs or Z-drugs, and most elderly patients were prescribed the same dose as younger adults. This ignores the US guidelines that state that older patients should receive half the standard adult dose and that doses should be limited to prevent addiction issues and severe side-effects [3]. Older adults are more susceptible to BZD or Z-drug toxicity, which might result in permanent sedation, falls, coma and/or death due to overdose [29]. High dosage prescriptions of BZDs or Z-drugs in KFCH suggest that elderly patients are vulnerable to poisoning by these medicines and that physicians are aiding their misuse. This agrees with a study in the UK [9], which reported that healthcare professionals are the most common cause of the misuse of BZDs and Z-drugs due to their inappropriate prescribing practice. 

Another significant problem is that approximately half of the prescriptions were not compliant with the recommended maximum duration of treatment (4–5 weeks). This finding closely mirrors previous research projects conducted by other researchers in the UK and Ireland, which report that BZDs and Z-drugs are often prescribed for extended periods [9,10,11,12], which increases the risk of dementia, especially with long-term use (more than three months) in elderly patients [30]. There is no knowledge about outcomes in patients who receive BZDs or zolpidem at KFCH long-term, especially regarding risks and side-effects.

The results of the present study showed that doctors did not review patients on long-term use of BZDs or zolpidem, the only Z-drug available in the KSA, once they began using the medication; this clarifies the continued prescription of these medicines for years by these physicians. This also clarifies the likelihood of misuse of BZDs or Z-drugs by patients, or the development of dependency or addiction to these drugs. It is possible, even probable, that most patients in the KSA are unaware of the risks or side effects of such medicines. In fact, during data collection, we noticed that patients often visited different clinics for the same medicine; these patients whose doctors fail to conduct a follow-up are at high risk of dependence and drug–drug interactions [29]. For example, evidence suggests that BZD use by patients with pulmonary disease might lead to respiratory depression [31]. Moreover, drugs lose their sleep-inducing efficiency with prolonged use [31]. 

This study had several strengths. To the best of our knowledge, this study is the first to explore the treatment of insomnia in the KSA. More importantly, it provides researchers with baseline information for further research. The study was also conducted in a major regional hospital (KFCH) in Jazan, one of the many hospitals run by the MOH. We feel that KFCH in Jazan is representative of other major hospitals of the MOH in the Kingdom. In other words, the practice identified here could be consistent with other major hospitals of the MOH and, thus, the findings might be extrapolated to them.

One of the study’s limitations is its retrospective nature; older records were still being copied by the Department of Medical Records from patients’ paper records at the time of the study, raising the possibility that the study’s information might be incomplete. Despite this, the study may present a reasonably accurate overview of current practice at KFCH. Another limitation is that it was conducted in a single hospital in the KSA. Further work is therefore needed to determine practices nationwide.

## 5. Conclusions

The present study shows that physicians at KFCH do not comply with the guidelines developed by the US [3,7] regarding the use of BZDs and Z-drugs to treat chronic, primary insomnia, and do not document indications for their use; the latter requires clarification. A second issue is that the non-pharmacological treatment form of insomnia (CBT-I) is not used in this major hospital. Therefore, this study’s essential recommendations are that initial treatments for patients with primary insomnia should be CBT-I and all physicians should be aware of its importance. Using non-pharmacological treatment methods might ameliorate and/or prevent drug misuse. In any case, prescribing practices related to the duration of treatment in the KSA do not conform to the US guidelines. To understand the basis for this occurrence, further research on the factors that guide diagnostic and treatment practices for insomnia in the Kingdom is required. Finally, the results show that patients using hypnotics for the long-term were not reviewed by their doctors. It is important for patients to attend weekly and monthly check-ups to ensure treatment process and the effects of medicines are determined in a timely manner. 

To overcome issues regarding the use of hypnotics in the KSA, the Saudi Ministry of Health should review physicians’ practices and the availability of medicines on the Drug List; offer training to prescribing physicians regarding best practice guidelines for managing insomnia; and introduce an electronic care plan that prompts prescribers to consider CBT-I, choose the correct drug, dose, duration, and review the patient’s medical history. The hospital’s computerized system should be improved and physicians should be trained to use it. Saudi standards and guidelines for insomnia treatment are required to govern the use and prescription of sleep medications. This will also help to provide long-term optimal patient care.

## Figures and Tables

**Table 1:** Data collection tool.

Patient Number
Prescribing physicians’ numbers
Patients who were prescribed these medications had clearly documented indications for use (YES/NO)
Patients received CBT-I as the initial treatment (documented) (YES/NO)
Patients were prescribed BZDs or Z-drugs if pharmacological treatments were considered (YES/NO)
Patients were initially prescribed the lowest licensed dosage (for adults or elderly) of BZDs or Z-drugs (YES/NO)
Patients were prescribed BZDs or Z-drugs for 4‒5 weeks (YES/NO)
Patients on long-term use of medicines were reviewed by their doctors every few weeks or monthly and then every 6 months (Documented) (YES/NO)
To treat chronic insomnia, anti-histamine or anti-histamine/ analgesic medicines were used (YES/NO)

**Table 2:** Number and percentage of the responses to the audit criteria.

Indicators	Records	Count Yes	Percentage %
Patients who received BZDs or Z-drugs had clearly documented indications for use	379	182	48
Patients received CBT-I as the initial treatment (documented)	307	0	0
Patients were prescribed BZDs or Z-drugs if pharmacological treatments were considered	307	182	59
Patients were initially prescribed the lowest licensed dosage of prescribed BZDs or Z-drugs	182	118	65
Patients were prescribed BZDs or Z-drugs for 4‒5 weeks	182	93	51
Patients on long-term use of medicines were reviewed by their doctors every few weeks or monthly, and then every 6 months	89	0	0
To treat chronic insomnia, anti-histamine or anti-histamine analgesic medicines were used	307	134	44

**Table 3:** Criteria followed by individual records.

Number of Criteria Met	Count	Percentage (%)
Met 0 criteria	13	4.2
Met 1 criterion	105	34.2
Met any 2 criteria	47	15.3
Met any 3 criteria	69	22.5
Met any 4 criteria	62	20.2
Met any 5 criteria	11	3.6
**Total**	**307**	**100**
